# Supplementary material for: Electrospun Nanofiber Membrane for Cultured Corneal Endothelial Cell Transplantation
Source: Bioengineering (Basel). 2024 Jan 5;11(1):54. doi: 10.3390/bioengineering11010054 (PMC10813262; doi:10.3390/bioengineering11010054)
Supplement: Supplementary file 1 [file bioengineering-11-00054-s001.zip › bioengineering-2788879-supplementary.pdf]

Supplementary Materials

Electrospun Nanofiber Membrane for Cultured Corneal Endothelial Cell Transplantation

Euisun Song [1,2], Karen M. Chen [3], Mathew S. Margolis [1], Thitima Wungcharoen [1,4], Won-Gun Koh [3] and David Myung [1,2,5,*]

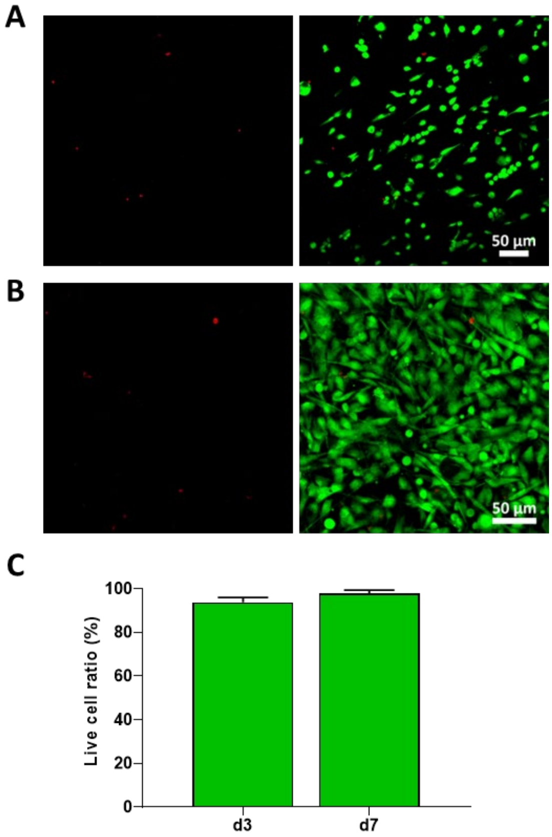

Figure S1. Cytotoxicity of gelNF. Live & Dead assay of the IHECE cultured on top of the gelNF membrane on days (A) 3 and (B) 7. Scale bars: 50 µm. (C) Quantified ratio of the live cell

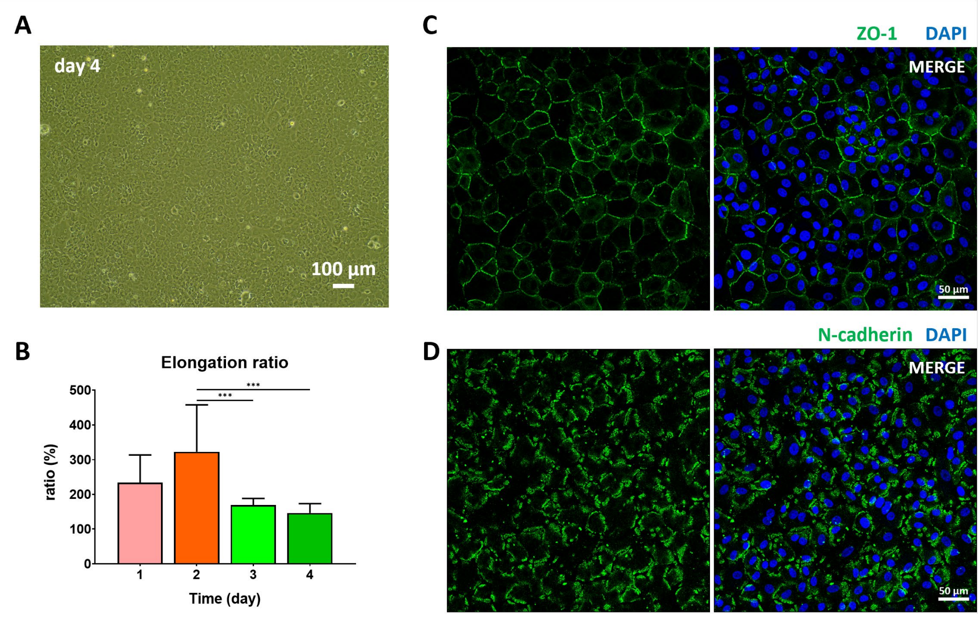

Figure S2. Isolation of primary rabbit corneal endothelial cell and characterization of shape and junctional protein expression. (A) Phase-contrast image of isolated PrCEC cultured on TCP on day 4. Scale bar: 100 µm. (B) Calculation of elongation ratio of PrCECs at different times (day). (C) Immunofluorescence staining of the ZO-1 (green) and nuclei (blue), and (D) N-cadherin (green) and nuclei (blue) on day 3. Scale bars: 50 µm.

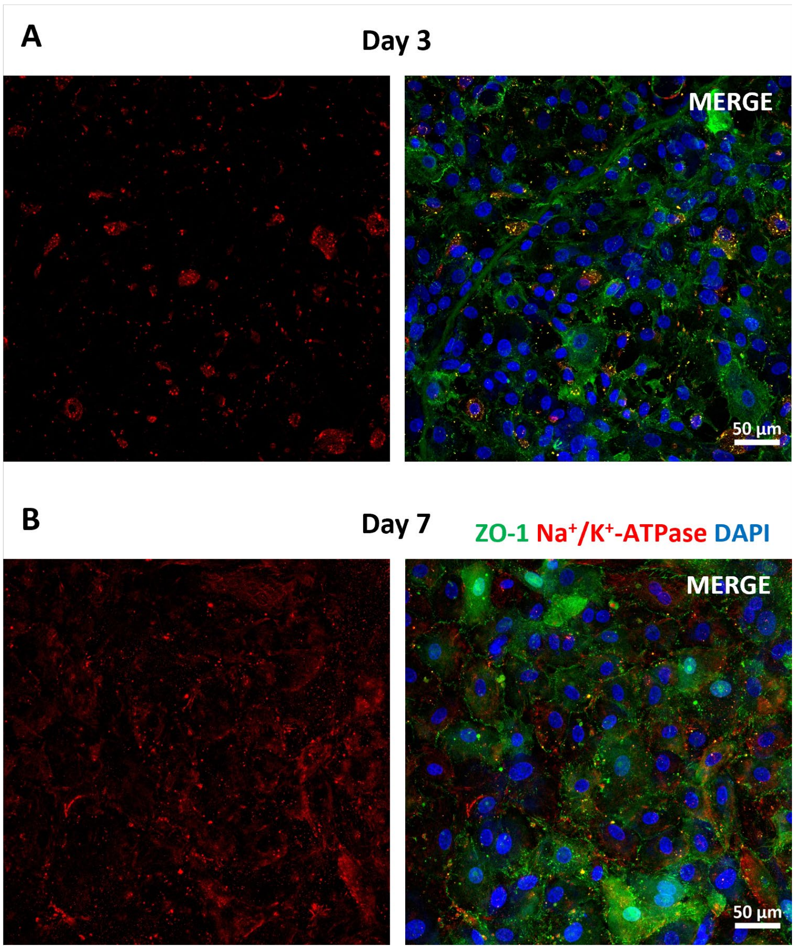

Figure S3. Functional protein expression of PrCECs cultured on top of the gelNF membrane. Immunofluorescence staining of the Na^+/K^+-ATPase (red), ZO-1 (green), and nuclei (DAPI) on (A) days 3 and (B) 7. Scale bars: 50 µm.
